# Expression of fibroblast specific protein-1 in pleural tuberculosis and its clinical biological significance

**DOI:** 10.1186/1477-7819-12-151

**Published:** 2014-05-20

**Authors:** Zhong-min Sun, Fei-yan Li, Lei Wang, Hong-yan Wang, Yuan Deng, Yan Yao

**Affiliations:** 1 Department of Respiratory and Critical Care Medicine, First Affiliated Hospital of Medical College of Xi'an Jiaotong University, No. 277 Yanta West Road, Xi'an, Shaanxi 710061, People’s Republic of China; 2 Department of Pathology, First Affiliated Hospital of Medical College of Xi'an Jiaotong University, No. 277 Yanta West Road, Xi'an, Shaanxi 710061, People’s Republic of China

**Keywords:** Pleural tuberculosis, Fibroblast specific protein-1, Pleural mesothelial cells, Pleural fibrosis

## Abstract

**Background:**

Fibroblast specific protein-1 (S100A4) is related with many fibrotic diseases, but its role in the pathogenesis of pleural fibrosis has not been fully elucidated. Then we aim to investigate the expression and effect of fibroblast specific protein-1 (S100A4) in pleural tuberculosis and, subsequently, pleural fibrosis.

**Methods:**

The expression of S100A4 in pleura was examined in 30 patients with pleural tuberculosis and 5 control (disease-free) patients by immunohistochemistry using the streptavidin-peroxidase (S-P) conjugated method.

**Results:**

The expression of S100A4 in pleura was mainly distributed in the nucleus and cytoplasm of fibroblasts and vascular endothelial cells, and the positive rate was 90.0% (27 out of 30 patients with pleural tuberculosis). There were no expressions of S100A4 in the control group. In the pleura of all 30 patients with pleural tuberculosis, S100A4 had a higher expression in the two- to eight-week duration of the disease.

**Conclusions:**

S100A4 plays an important role in the phenotypic transformation of pleural mesothelial cells and the development of pleural fibrosis.

## Background

Incidences of pleural fibrosis are not rare and may cause restrictive ventilation dysfunction, which significantly reduces patients’ quality of life. Pneumonia, parapneumonic effusion, empyema, pulmonary tuberculosis, and pleural tuberculosis are the most common causes of pleural fibrosis, especially pleural tuberculosis. It is known that pleural mesothelial cell transition into myofibroblasts (MMT) plays an important role in the pathogenesis of pleural fibrosis. However, the pathogenesis of pleural fibrosis has not been fully elucidated yet.

Fibroblast specific protein-1 (S100A4) is a member of the S100 family of proteins containing 2 EF-hand calcium-binding motifs, known as a metastasis-associated protein, whose chromosomal rearrangements and altered expression have been implicated in tumor metastasis [1]. However, recent studies have linked S100A4 to some fibrotic diseases [2], and it is reported that S100A4 contributes to the pathogenesis of oral submucous fibrosis [3]. There is also research evidencing that S100A4 modulates the p53 function in fibroblasts to mediate myocardial interstitial fibrosis [4]. So far, the role of S100A4 in the pathogenesis of pleural fibrosis resulting from pleural tuberculosis has not been reported.

Our study was designed to investigated the expression of S100A4 in pleural tuberculosis and analyze its relationship to the clinical course of pleural tuberculosis, aiming to explore the potential mechanism of pleural fibrosis caused by pleural tuberculosis.

## Methods

### Source of specimen

The study was approved by the Research Ethics Committee of the First Affiliated Hospital of the Medical College of Xi'an Jiaotong University (People’s Republic of China) and written informed consent was obtained from all patients before the procedure.

Paraffin-embedded pleural tissue specimens from 35 cases, among them, 30 cases were from pleural tuberculosis patients, and the other 5 cases were from normal pleural tissue. The samples are got by the percutaneous needle pleural biopsy or surgical operation in First Affiliated Hospital of Medical College of Xi'an Jiaotong University from June, 2011 to June 2012.

Among the 30 patients with pleural tuberculosis, 14 cases are male, and 16 cases are female, aged between 14 to 53 years old. 5 normal cases included 3 males and 2 females, age ranged 13 to 40 years old.

### Methods and reagents

Paraffin-embedded tissue sections were made, roughly 4 μm in size. In order to review the diagnosis, one section was processed using S100A4 dyeing and the other was processed using hematoxylin and eosin dyeing.

Rabbit anti-human S100A4 polyclonal antibody (Neomarker, Fremont, CA, USA) is used for S100A4 dyeing.l. The broad spectrum allergic S-P kit and Diaminobenzidine (DAB) chromogenic liquid were purchased from Fuzhou Mai'xin biotechnology company (Fuzhou, China). S100A4 was detected referring to the S-P immunohistochemistry kit instructions. A known S100A4-positive tonsil slice was made as a positive control, and phosphate buffer solution (PBS) used instead of the primary antibodies for a negative control.

### Double-blind method to view the slices

Yellow granules in cytoplasm or in the nucleus were considered as positive cells. The Fromwitz comprehensive scoring method [5] for protein accumulation was slightly modified to process a semi-quantitative analysis. Under a high magnification (10 × 40), 10 different fields were randomly selected with 100 cells in each field. We then calculated the positive cell percentage (positive cells/total number of cells). Positive percentage scoring was as follows: ≤ 5% for 0 point, 6 to 25% for 1 point, 26 to 50% for 2 points, 51 to 75% for 3 points, >75% for 4 points. We applied a tinting strength score for the staining of positive cells: light brown for 1 point, brown for 2 points, and deep brown for 3 points. The process comprehensive scoring (positive percentage score and tinting strength score are calculated respectively, then summed.) was as follows: 0 to 1 point was negative (−), 2 to 3 points was weakly positive (+), 4 to 5 points was moderately positive (+ +), and 6 to 7 points was strong positive (+ + +).

### Statistical analysis

SPSS13.0 statistical software (SPSS Inc, Chicago, IL,USA) was used perform the statistical analysis. The rank-sum test (Kruskal-Wallis test), and pairwise comparison with the Bonferroni method was used (*P* <0.05 was considered to be statistically significant). One-way analysis of variance (ANOVA), and pairwise comparison using the least significance difference (LSD) method was performed (*P* <0.05 was considered to be statistically significant).

## Results

### S100A4 expression orientation

**Figure 1:**
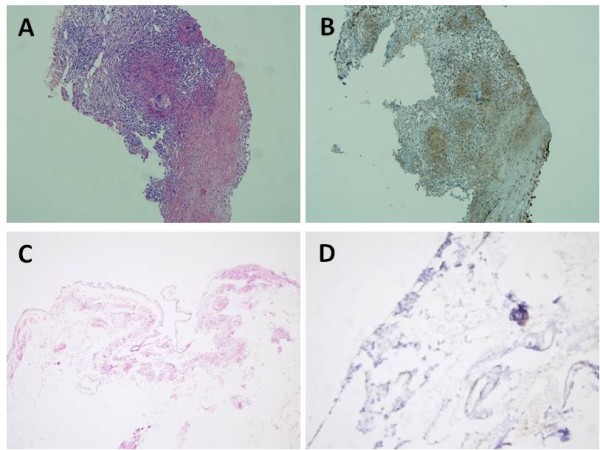
**The expression of S100A4 in pleural tissue. (A)** HE stained of tuberculous pleurisy (10 × 10). **(B)** S100A4 protein expression in tuberculous pleurisy in cell cytoplasm and nucleus, stained by S-P method (10 × 10). The S100A4 positive cells are brown. **(C)** HE stained of normal pleural tissue (10 × 10). **(D)** No brown cells were observed. S100A4 protein is not expressed in normal pleural tissues, stained by S-P method (10 × 10).

S100A4-positive expression in pleural tissue showed as claybank granules under high magnification. The results revealed that S100A4 was mainly located in the cytoplasm and nucleus of interstitial fibroblasts and vascular endothelial cells. The positive cells were unevenly distributed, with often focal or patchy distribution. The total positive rate was 90.0% (27 out of 30 patients with pleural tuberculosis). The expression of S100A4 in normal pleural tissues was negative (showed as Figure 1).

### The relationship between S100A4 expression and the course of pleural tuberculosis

The expression of S100A4 is closely associated with the clinical course of pleural tuberculosis (showed in Table 1). In cases for which the clinical course were between two to eight weeks, the expressions were all positive and the comprehensive score was highest, compared with other patients group.

**Table 1:** The relationship between S100A4 expression and the course of pleural tuberculosis

**Course**	**Patients number**	**S100A4 expression comprehensive score**				**P value**
		**-**	**+**	**++**	**+++**	
<2 weeks	11	3	5	1	2	0.09^a^
2-8 weeks	13	0	1	7	5	0.01^b^
>8 weeks	6	0	6	0	0	

^a^:<2 weeks vs. 2-8 weeks; ^b^: 2-8 weeks vs. >8 weeks. Both are calculated by rank-sum test.

## Discussion

Our study found that S100A4 was significantly expressed in pleural tuberculosis, and was closely associated with the clinical course of pleural tuberculosis, while it did not express in normal pleural tissue.

In China, tuberculous pleural effusion accounts for about 50% of the total pleural effusion. Although regular anti-tuberculosis treatment is available, there are still 30 to 50% of tuberculous pleural effusion patients progressing to pleural thickening and adhesion [6]. Pleural fibrosis can have a certain degree influence on the body, some may cause fibrothorax and lead to respiratory dysfunction. At present, there is no effective method to cure pleural fibrosis. In recent years, the mechanism of pleural thickening and adhesion has been controversial. Some researchers present that the pleural thickening degree is associated with elevated protein and adenosine deaminase (ADA) in pleural effusion [7]. While some researchers oppose this view, and instead present that the decrease of PH and glucose in the thoracic liquid is associated with pleural thickening [8]. Studies have also shown that the pleural mesothelium cells (PMCs), cytokines, and fibrinolytic and/or coagulation system play an important role in the pathogenesis of pleural fibrosis [9,10].

PMCs can respond to some signals in the microenvironment and secrete glycosaminoglycan and surfactant to reduce the friction between visceral pleura and parietal pleura. Besides this, PMCs also have other biological behaviors to avoid pleural thickening and adhesion, for example, participating in fluid transfer, particles passing in and out of the serous cavity, inflammation reaction, growth factors and collagen releasing to repair damage, and protease and fibrinolytic enzyme-releasing. Huggins *et al.* presented that the response of PMCs to injury and its ability, along with the basement membrane, to maintain its integrity is vital in determining whether there is normal healing or pleural fibrosis [11]. The research of Antony [12] showed that fibroblast growth factor (bFGF) produced by PMCs can affect pleural fibrosis. Nathalie [13] transferred TGF-β1 into the pleural mesothelium cell of rats by adenovirus gene transfection, which induced the occurrence of pleural fibrosis and caused serious lung capacity limits. The results also showed that pleural fibrosis was associated with PMCs phenotypic transformation to α-SMA-positive myofibroblasts, called mesothelial-fibroblastoid transformation.

TGF-β is an important pro-fibrosis factor and plays an important role in fibrosis of many organs. Studies have shown that TGF-β can induce the occurrence of epithelial-mesenchymal transition (EMT) [14]. Lung tissue transient overexpression of TGF-β1 can induce pulmonary fibrosis [15]. TGF-β can also lead to the occurrence of pleural fibrosis by increasing the production of extracellular matrix and decreasing its degradation [16]. Myofibroblasts are a cell type that between fibroblasts and smooth muscle cells, it retains the biological characteristics of fibroblasts to synthesize collagen, which is the main source of extracellular matrix in organs fibrosis, and the contraction characteristics of smooth muscle cells. Kim *et al.* showed that pleural mesothelial cells treated with TGF-β1 would lose the epithelial morphology and gain epithelial and mesenchymal characteristics. This shows that the pleural mesothelial cells in pleural tuberculosis participated in pleural fibrosis through EMT [17].

In 1995, S100A4 was found to especially be expressed in fibroblasts and named it fibroblast specific protein-1 [18]. S100A4 is a calcium-binding protein which has an EF double helix structure, and together with another 19 members, composes the S100 family. It participates in signal transduction inside and outside the cell, cell proliferation and differentiation, intercellular adhesion and cell movement, and many other physiological processes. For more than 10 years, many studies have shown that S100A4 plays an important role in tumor metastasis [19]. Recent studies have also shown that TGF-β1 can regulate the expression of S100A4, and cancer cells invasion mediated by TGF-β1 could be inhibited by S100A2 silence, indicating that S100A4 might be the molecular target of TGF-β1 in tumor metastasis [20].

## Conclusions

Our study found that S100A4 was positively expressed in pleural tuberculosis, mainly in the fibroblasts and not in normal pleural tissue. This result suggests that pleural interstitial cells may increase in pleural tuberculosis, consistent with prior research conclusions. Therefore, S100A4 may play an important role in the phenotype transformation of pleural mesothelium cells and pleural fibrosis. But whether pleural mesenchymal cells derive from epithelial cells via EMT during the process of pleural fibrosis, and the potential role of TGF-β and S100A4 in this warrant further study.

## Abbreviations

S100A4: fibroblast specific protein-1; MMT: mesothelial cell transition into myofibroblasts; DAB: Diaminobenzidine; PBS: phosphate buffer solution; ANOVA: One-way analysis of variance; LSD: least significance difference; ADA: adenosine deaminase; PMCs: pleural mesothelium cells; bFGF: fibroblast growth factor; EMT: epithelial-mesenchymal transition.

## Competing interests

The authors declare that they have no competing interests.

## Authors’ contributions

Z-mS, F-yL, LW, H-yW, YD and YY carried out the experiments, Z-mS designed the study, and Z-mS and LW prepared the manuscript. All authors read and approved the final manuscript.
